# Supplementary material for: Neuroprotective Effects of an Aqueous Extract of Forsythia viridissima and Its Major Constituents on Oxaliplatin-Induced Peripheral Neuropathy
Source: Molecules. 2019 Mar 25;24(6):1177. doi: 10.3390/molecules24061177 (PMC6471886; doi:10.3390/molecules24061177)

Supplementary Material

Neuroprotective Effects of an Aqueous Extract of *Forsythia Viridissima* and Its Major Constituents on Oxaliplatin-Induced Peripheral Neuropathy

Jin-Mu Yi, Sarah Shin, No Soo Kim and Ok-Sun Bang *

Clinical Medicine Division, Korea Institute of Oriental Medicine, Daejeon 34054, Republic of Korea; jmyi@kiom.re.kr (J.-M.Y.); s.sarah@kiom.re.kr (S.S.); nosookim@kiom.re.kr (N.S.K.)

* Correspondence: osbang@kiom.re.kr; Tel.: +82-42-8689353

Supplementary Figure 1. Comparison of DAD spectra between major peaks of EFVF and their STDs.

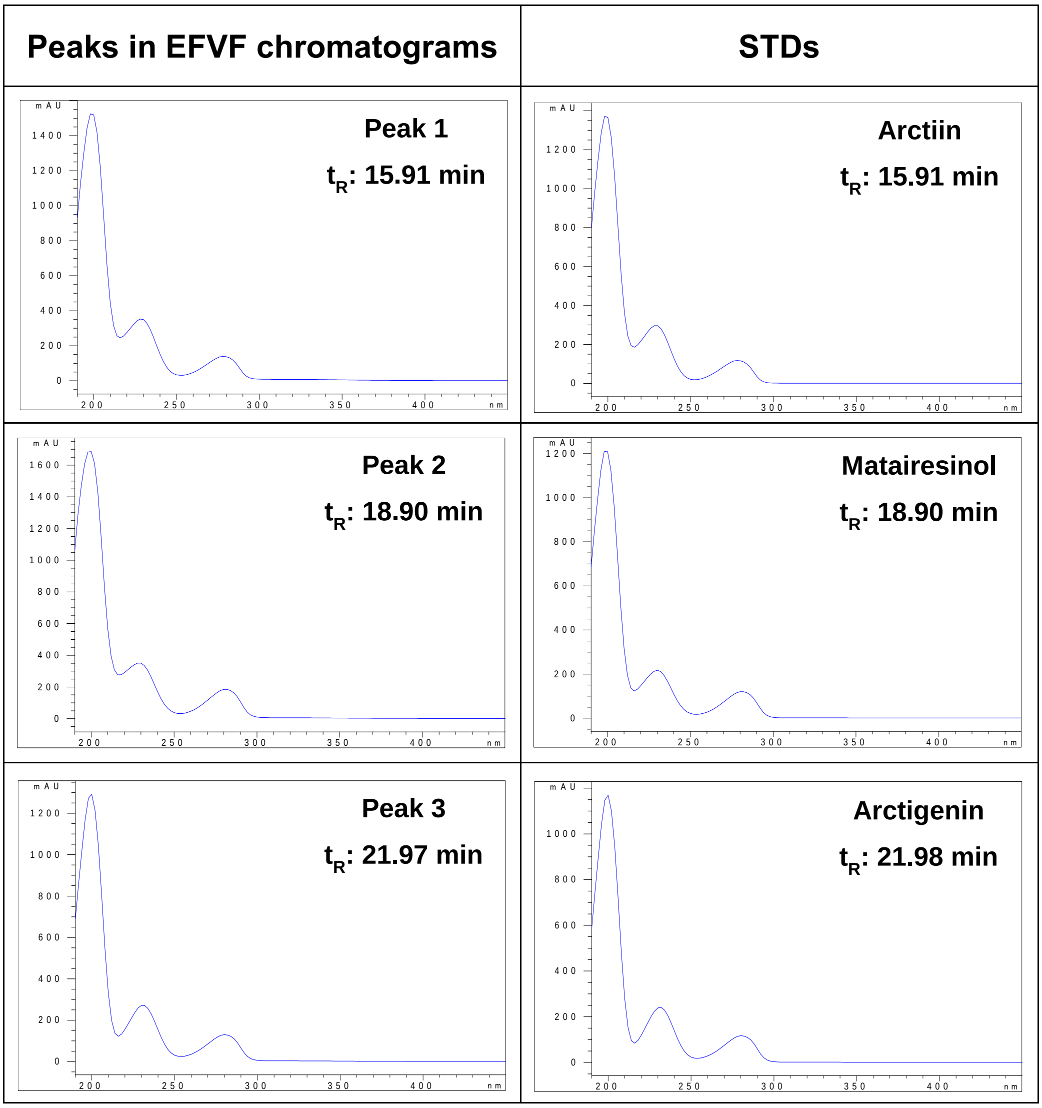

Supplement: Supplementary file 1 [file molecules-24-01177-s001.pdf]
